# Professor George T. Barker

**Published:** 1878-03

**Authors:** 


### OBITUARY.

---

*Dr. George T. Barker.*—Died, of typhoid fever, January 10th, 1878, George T. Barker, D. D. S., in the forty-second year of his age.

Dr. Barker was born in Poughkeepsie, Dutchess Co., N. Y., March 26th, 1835, where he spent his youth and received his early education. At the age of nineteen he removed to Baltimore, Md.. and commenced the study of dentistry with Dr. Samuel Townsend, with whom he remained a year or more. In the year 1866 he removed to Philadelphia, and became an office-student with Dr. Edward Townsend. He attended lectures at the Pennsylvania College of Dental Surgery, and graduated from that institution in 1859 ; was elected professor of Dental Pathology and Therapeutics in the autumn of 1862, which position he occupied until the time of his death. He was elected Dean of the Faculty in March, 1876, which office he retained until March, 1877.

Dr. Barker was one of the original editors of the *Denta Times ;* was the author of a work entitled "Instructions in Nitrous Oxide ;" was a contributor to the various dental journals; was a member of the American Dental Association, a member o the Pennsylvania State Dental Society, a member of the Pennsylvania Association of Dental Surgeons, etc. On the 26th day of April, 1860, Dr. Barker was united in marriage to Susan

Ridgway, daughter of Thomas Ridgway, Esq., of Philadelphia. A son and daughter survive him as the result of this union.

Dr. Barker was a member of the Society of Friends, and also belonged to the order of Masons.

Ourselves marching to the eternal world, it is but fitting that we loiter for a moment on the busy highway of life to record the virtues of our fallen comrade.

Dr. Barker was a man of positive character, and, like all men thus constituted, his likes and dislikes were easily recognizable and his motives liable to be misunderstood. Yet a prolonged acquaintance assured one of his frankness of disposition and his willingness to make suitable amends where wrong impressions had been conveyed.

He was a zealous worker in his profession, ever ready to defend its dignity and its advancement. As a teacher, he was able, conscientious and popular, and for fifteen years was instrumental in perfecting young men in the branches which he taught with more than ordinary ability.

As a practitioner, he was successful, enjoying the confidence of many and securing to himself a large and lucrative practice.

As a citizen, he was loyal and brave, frequently defending in public the rights of the oppressed, and seeking to alleviate the sufferings of the unfortunate. For years it had been his practice to spend evenings in our city hospitals and institutions of charity, reading and reciting popular stories for the pleasure and entertainment of the inmates. Eminently social in disposition and lively in manner, he attracted to himself a large circle of friends and acquaintances, who will profoundly lament his untimely death.

If I may venture to enter the sacred precincts of home and speak of him as husband and father, it is in that capacity that his character shone forth with greatest brilliancy. Dr. Barker's home-life was beautiful. When he crossed the threshold of home he dropped the cares and perplexities of business and entered into joyous sympathy with wife and children. His social instincts made him a companion to his family, by whom his untimely removal will be most keenly felt.

